# Sas-4 Colocalizes with the Ciliary Rootlets of the *Drosophila* Sensory Organs

**DOI:** 10.3390/jdb9010001

**Published:** 2021-01-05

**Authors:** Veronica Persico, Giuliano Callaini, Maria Giovanna Riparbelli

**Affiliations:** 1 Department of Life Sciences, University of Siena, Via Aldo Moro 2, 53100 Siena, Italy; persico@student.unisi.it (V.P.); riparbelli@unisi.it (M.G.R.); 2 Department of Medical Biotechnologies, University of Siena, Via Aldo Moro 2, 53100 Siena, Italy

**Keywords:** *Drosophila*, sensory organs, rootlets, rootletin, Sas-4

## Abstract

The *Drosophila* eye displays peculiar sensory organs of unknown function, the mechanosensory bristles, that are intercalated among the adjacent ommatidia. Like the other *Drosophila* sensory organs, the mechanosensory bristles consist of a bipolar neuron and two tandemly aligned centrioles, the distal of which nucleates the ciliary axoneme and represents the starting point of the ciliary rootlets. We report here that the centriole associated protein Sas-4 colocalizes with the short ciliary rootlets of the mechanosensory bristles and with the elongated rootlets of chordotonal and olfactory neurons. This finding suggests an unexpected cytoplasmic localization of Sas-4 protein and points to a new underscored role for this protein. Moreover, we observed that the sheath cells associated with the sensory neurons also display two tandemly aligned centrioles but lacks ciliary axonemes, suggesting that the dendrites of the sensory neurons are dispensable for the assembly of aligned centrioles and rootlets.

## 1. Introduction

*Drosophila melanogaster* has two main kinds of sensory organs that display some variations with respect to their specific sensory function [1]. Chemosensory organs usually consist of elongated bristles holding the ciliated region of the sensory neurons. Mechanosensory organs lack cuticular extensions and the ciliated end of the sensory neurons is anchored to the dendritic cap, an apical cuticular dome [2]. Despite this different morphology the sensory organs share a common module consisting of supporting cells, the sensory neuron, and a modified primary cilium. The scaffolding structure of the ciliary projections is a microtubule based axoneme that nucleates from the apical end of the distal centriole, here acting as a basal body. A second centriole, the proximal one, is coaxial to the distal. Despite this unusual disposition, the distal and the proximal centrioles are retained homologous to the mother and the daughter centrioles of somatic cells on the basis that the primary cilium is organizes in vertebrate cells by the mother centriole. This aspect is unclear since the early biogenesis of the centrioles in sensory organs is still unknow. Moreover, both the parent centrioles can nucleate a ciliary axoneme during male gametogenesis [3]. However, only the proximal centriole in sensory neurons expresses centrobin, a protein that is usually restricted to the daughter centriole in *Drosophila* somatic cells [4,5]. Accordingly, ectopically expressed centrobin in sensory organs results in centrioles unable to nucleate ciliary axonemes supporting the role of this protein in affecting the fate and function of the parent centrioles [6].

A common feature of the sensory organs is the presence of ciliary rootlets, fibrous cross-striated cytoskeletal structures, which arise close to the base of the distal centriole and extend proximally. The distal centrioles seem to play a main role in the proper assembly of the ciliary rootlets. Accordingly, sensory neurons of fly mutants for centriole assembling proteins lack normal rootlets or have a few rootlet-like structures [7]. Although, the function of the rootlets is unclear, depletion of these structures in *Drosophila*, impaired the function of the sensory neurons resulting in dramatic behavior defects [7].

Rootletin was firstly identified as a structural component of the ciliary rootlets in murine photoreceptor cells [8] and is the major component of vertebrates [9,10,11] and *Drosophila* ciliary rootlets [7,12]. In proliferating mammalian cells when cilia are not assembled, the rootletin is also part of the tether that links mother and daughter centrioles during the cell cycle [13,14]. A role of this protein in parent centriole connection has not been described in *Drosophila*. However, ectopically expressed rootletin localizes asymmetrically at the base of the mother centrioles in primary *Drosophila* spermatocytes and neuroblasts [7].

Besides the mechanosensory and chemosensory organs, *Drosophila* harbors another peculiar sensory organ, the interommatidial bristles, which specific function is unknown. The interommatidial bristles are intercalated among the adjacent ommatidia of the developing and adult compound eye and have the usual organization of the sensory organs [15], originating from a typical mechanosensory cell lineage [16].

By looking at the localization of centriole associated proteins in the interommatidial bristles, we find an atypical distribution of Sas-4. The Sas-4 protein plays a central role in the structural organization of the centrioles as it regulates the polymerization of the centriolar microtubules and their stability [17]. Sas-4 is, also, essential for pericentriolar material tethering during centrosome maturation [18,19] and appears as a negative regulator of ciliary length independent of its role in centrosome biogenesis [20]. Moreover, Sas-4 interacts with centriolar microtubules by inhibiting catastrophes and promoting rescues to ensure slow assembly and modulate centriole size and dimension [21]. Hence, being a structural protein involved in the organization of the tubule wall [22,23,24,25], Sas-4 should be restricted to the centriole itself. However, we found that the localization of Sas-4 was not restricted to the tandemly aligned centrioles but was also detected along the ciliary rootlets. This unusual colocalization of Sas-4 with the ciliary rootlets was also observed in the chordotonal and olfactory neurons of the antennae. Two tandemly aligned centrioles and a reduced ciliary rootlet have been also found in the sheath cells that surround the sensory neurons of the interommatidial bristles. This suggests that the dendrites of the sensory neurons are dispensable for the assembly of aligned centrioles and rootlets.

## 2. Materials and Methods

### 2.1. Drosophila Strains

Rootletin-GFP [7] flies were kindly provided by L. Kovacs; the Unc-GFP [26] stock was kindly provided by T. Megraw; Sas-4-GFP [27] flies were a gift of J. Gopalakrishnan; The Sas-4 mutant allele [28] was a gift of J. Raff. All flies were reared on a standard cornmeal medium [29] at 24 °C under a 12 h light 12 h dark cycle. OregonR stock was used as the wild-type. 

To obtain samples at the similar developmental stages we collected eggs three times for 20 min each from 4–5-day-old flies. Egg precollection needs to have fertilized oocytes at the same stage, since females store fertilized eggs for different periods of time. After discarding the first eggs, we made a new collection for 1 h and leaved the eggs to develop in plastic vials containing the food. We examined for each strain 25 eye imaginal discs from pupae at 25 h and 45 h after puparium formation (APF). 

### 2.2. Antibodies

We used the following antibodies: Mouse anti-acetylated tubulin (1:100; Sigma-Aldrich, St. Louis, MO, USA); mouse anti-Sas-4 (1:200; [30]; rabbit anti-centrobin (Cnb) (1:200; [31]; chicken anti-Pericentrin-like protein (Plp) (1:1500; [32]). The secondary antibodies used (1:800) were Alexa Fluor-488-, Alexa Fluor-555- and Alexa Fluor-647-conjugated anti-mouse-IgG, anti-rabbit-IgG, anti-chicken-IgG were obtained from Invitrogen.

### 2.3. Immunofluorescence Staining

Eye imaginal discs from both male and female pupae at 25 h and 45 h APF were dissected in phosphate-buffered saline (PBS) and fixed in cold methanol for 10 min at −20 °C. For antigen localization, the samples were washed for 20 min in PBS and incubated for 1 h in PBS containing 0.1% bovine serum albumin (PBS-BSA, from Sigma-Aldrich) to block nonspecific staining. The samples were incubated overnight at 4 °C with the specific antisera in a humid chamber. After washing in PBS-BSA, the samples were incubated for 1 h at room temperature with the appropriate secondary antibodies. Imaginal discs and antennae were mounted in small drops of 90% glycerol in PBS. 

### 2.4. Transmission Electron Microscopy

Eye imaginal discs were isolated from pupae at 45 h APF and transferred in 2.5% glutaraldehyde buffered in PBS overnight at 4 °C. Samples were then rinsed in PBS and post-fixed in 1% osmium tetroxide in PBS for 2 h at 4 °C. The material was washed in PBS, dehydrated in a graded series of ethanol, embedded in a mixture of Epon-araldite resin, and then polymerized at 60 °C for 48 h. Thin sections (40–50 nm thick) were obtained with a Reichert Ultracut E ultramicrotome equipped with a diamond knife, mounted upon copper grids, and stained with uranyl acetate and lead citrate. Samples were observed with a Tecnai Spirit Transmission Electron Microscope (FEI) operating at 100 kV and equipped with a Morada CCD camera (Olympus).

### 2.5. Image Acquisition

Images were taken by an Axio Imager Z1 microscope (Carl Zeiss, Jena, Germany), using a 100× objective, equipped with an AxioCam HR cooled charge-coupled camera (Carl Zeiss). Gray-scale digital images were collected separately and then pseudocolored and merged using Adobe Photoshop 5.5 software (Adobe Systems, San Jose, CA, USA).

## 3. Results

**Figure 1:**
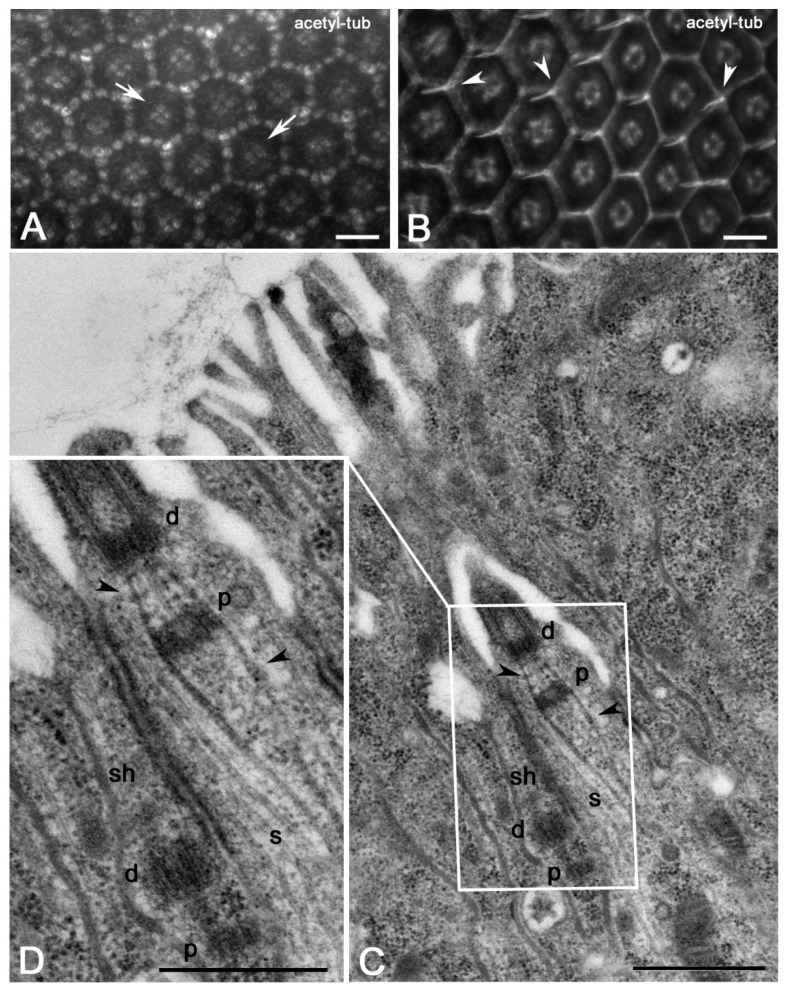
Surface view of the wild-type *Drosophila* retina at 25 h (**A**) and 45 h (**B**) after puparium formation (APF): At 25 h APF the interommatidial cells are disposed in a precise hexagonal pattern around the four cone cells of each ommatidium (arrows); after 45 h APF the interommatidial cells flattened and distinct mechanosensory bristles are visible at the apex of each ommatidial unit (arrowheads). Longitudinal sections of a wild-type mechanosensory bristle (**C**) and magnification of its basal region (**D**): tandemly aligned centrioles are present in both the sensory (s) and sheath (sh) cells; (d) distal and (p) proximal centrioles, arrowheads point to rootlets. Scale bars: 10 µm (**A**,**B**), 500 nm (**C**,**D**).

The eye imaginal discs of early pupae consist of a thin epithelium in which the differentiating ommatidia are separated by a matrix of unpatterned interommatidial cells arranged in several rows [33,34]. The eye pattern formation continues during pupal stages and the interommatidial cells gradually reduce to single rows arranged in precise hexagonal structures around the photoreceptor cells (Figure 1A). Some of the interommatidial cells are then removed by programmed cell death to get to the final required cell number [15] and the remnant cells flattened (Figure 1B). A mechanosensory bristle appeared at every other apex of each ommatidial unit (Figure 1B). The bristles are crossed by a structured axoneme nucleated by a distal centriole that is coaxial with a proximal one (Figure 1C; [35]). The basis of the distal centriole is the starting point of striated filamentous structures, the ciliary rootlets, that surround the proximal centrioles and project towards the basal region of the cell (Figure 1C,D). Surprisingly, we found that all the sheath cells in suitable orientation (n = 24) examined by electron microscopy from five eye pupal imaginal discs at 45 h APF displayed a pair of tandemly aligned centrioles and very short rootlets. However, the distal centrioles of the sheath cells lack a ciliary axoneme (Figure 1D). This suggests that the distal centrioles of the sheath cells do not convert to basal bodies. 

**Figure 2:**
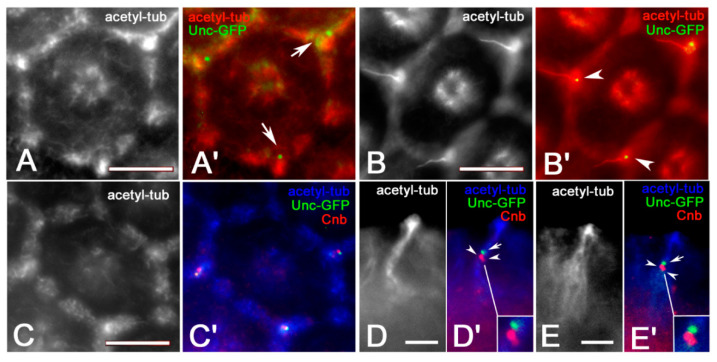
Localization of Unc-GFP in ommatidia at 25 (**A**,**A′**) and 45 (**B**,**B′**) hours APF: Only one Unc-GFP spot is associated with the bristle mother cells (arrows, **A′**) and with the basis of the interommatidial bristles (arrowheads, **B′**). (**C**,**C′**) The Unc-GFP spots do not overlap the centrobin (Cnb) staining in the bristle mother cells. (**D**,**D′**,**E**,**E′**) Longitudinal view of growing bristles showing one distal Unc signal (arrows) and two proximal centrobin spots (arrowheads). Scale bars: 5 µm (**A**–**C**), 2.5 µm (**D**,**E**).

To verify this possibility, we asked if only the centrioles that organize the ciliary axonemes of the interommatidial bristles expressed Unc-GFP, a distinctive protein of the *Drosophila* basal bodies [26]. One Unc-GFP spot was, indeed, associated with the bristle mother cells before axoneme assembly (Figure 2A,A′) or with the proximal region of the elongating mechanosensory bristle (Figure 2B,B′). To confirm that the centriole associated with the ciliary axoneme was the distal one, we look at the distribution of centrobin, a good marker for the daughter centrioles in *Drosophila* [4,5]. We observed that one centriole at the base of the forming mechanosensory bristle was stained for centrobin (Figure 2C,C′). However, when the elongating bristles were imaged sideways (Figure 2D,E), we find one distal bright Unc-GFP dot and a pair of proximal centrobin dots (Figure 2D′,E′). This suggests that only the distal centriole involved in the nucleation of the axoneme express Unc-GFP, whereas the proximal centrioles of the sheath cells and neurons express centrobin.

**Figure 3:**
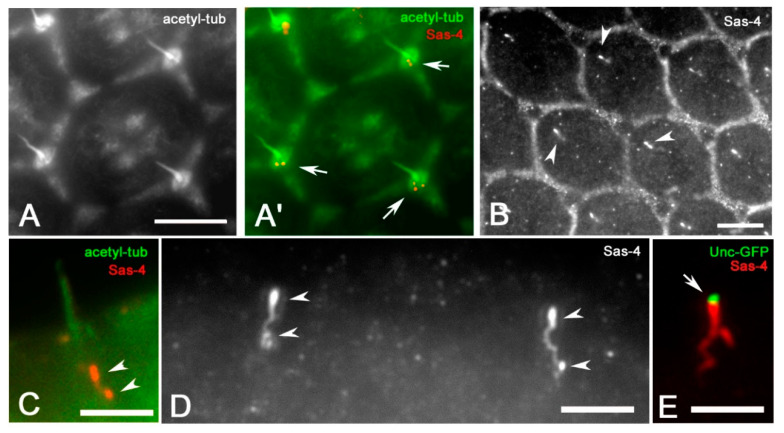
Surface view of wild-type ommatidia at 45 h APF (**A**,**A′**) showing Sas-4 spots (arrows) at the basis of the bristles. (**B**) Lower focus level of ommatidia at 45 h APF showing elongated Sas-4 filaments (arrowheads). (**C**) Lateral view of a bristle and the associated Sas-4 filaments (arrowheads). (**D**) Magnification of paired Sas-4 filaments (arrowheads) from two adjacent ommatidia. (**E**) The Unc-GFP signal is only associated with the apical end of the more elongated Sas-4 filament. Scale bars: 5 µm (**A**,**B**), 2.5 µm (**C**–**E**).

To unambiguously detect the centrioles of the mechanosensory bristles we checked the localization of the conserved centriole specific core protein Sas-4, which provides a link between the cartwheel and the microtubule wall [36,37] and may represent a bona fide marker of centrioles in the developing eye. Surface views of the *Drosophila* retina showed two or more Sas-4 spots at the base of each interommatidial bristle (Figure 3A,A′). However, looking at lower focal planes, we find that the antibody against Sas-4 recognized more complexes structures within each ommatidium (Figure 3B). Lateral views showed that these structures were localized below the bristle (Figure 3C) and appeared as paired bright filaments of different length that emerged from two separated centrioles (Figure 3D). 

Remarkably, the Unc-GFP signal was only found at the apical end of the more elongated Sas-4 filament (Figure 3E) suggesting that this filament is associated with the distal centriole that is involved in the formation of the ciliary axoneme. 

**Figure 4:**
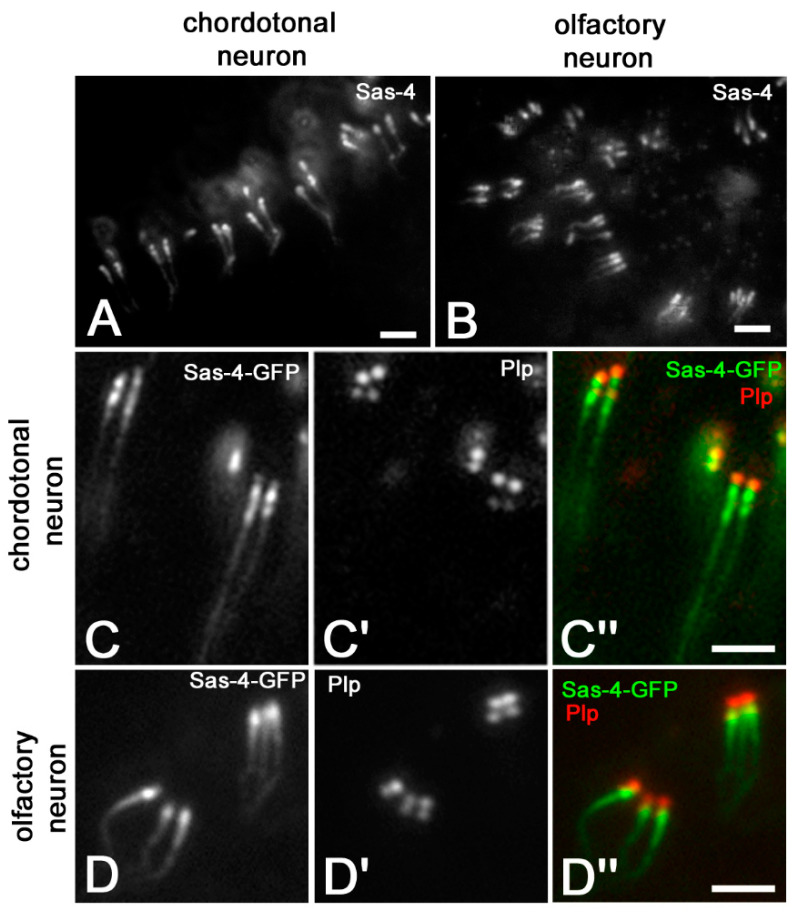
Localization of Sas-4 in wild-type chordotonal (**A**) and olfactory (**B**) neurons and Sas-4-GFP expression in chordotonal (**C**,**C′**,**C″**) and olfactory (**D**,**D′**,**D″**) neurons; centrioles are underlined by Plp localization. Scale bars: 5 µm.

The above observations suggest that the fluorescence staining found with the Sas-4 antibody could correspond to the complex consisting of the distal and proximal centrioles and the ciliary rootlets. To verify this possibility, we examined the sensory antennal organs. The second and third antennal segments have, indeed, sensory neurons that display developed ciliary axonemes, tandemly aligned centrioles and very elongated ciliary rootlets thus representing good model in which to examine an eventual ectopic localization of the Sas-4 protein. Like to the mechanosensory bristles, the Sas-4 antibody recognized in both chordotonal (Figure 4A) and olfactory (Figure 4B) organs the parent centrioles and distinct filamentous structures. 

To exclude that the Sas-4 signal at the base of the ciliary structures was due to a cross-reactivity of the monoclonal antibody, we analyzed a *Drosophila* strain expressing the Sas-4 protein conjugated with GFP [27]. The localization of the endogenous Sas-4 protein on chordotonal (Figure 4C,C″) and olfactory (Figure 4D,D″) organs was the same observed with the monoclonal antibody. Sas-4-GFP also localized to centrioles, as confirmed by the expression of the pericentrin-like protein (Plp) (Figure 4C′,C″,D′,D″). 

**Figure 5:**
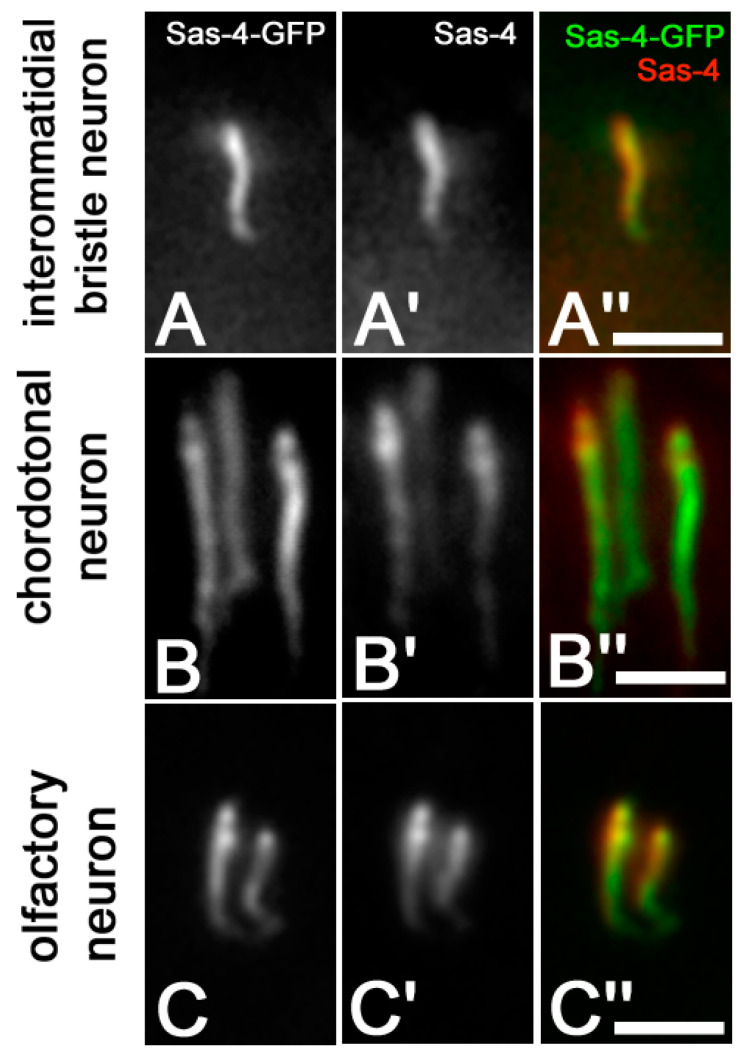
Colocalization of Sas-4-GFP signal and anti-Sas-4 stain in the interommatidial bristle (**A**–**A″**), chordotonal (**B**–**B″**) and olfactory (**C**–**C″**) neurons from pupae of 45 h APF. Scale bars: 15 µm.

Moreover, the Sas-4-GFP signal overlapped the labelling obtained with the monoclonal anti-Sas-4 antibody in the eye imaginal discs (Figure 5A–A″), chordotonal (Figure 5B–B″) and olfactory (Figure 5C–C″) organs.

**Figure 6:**
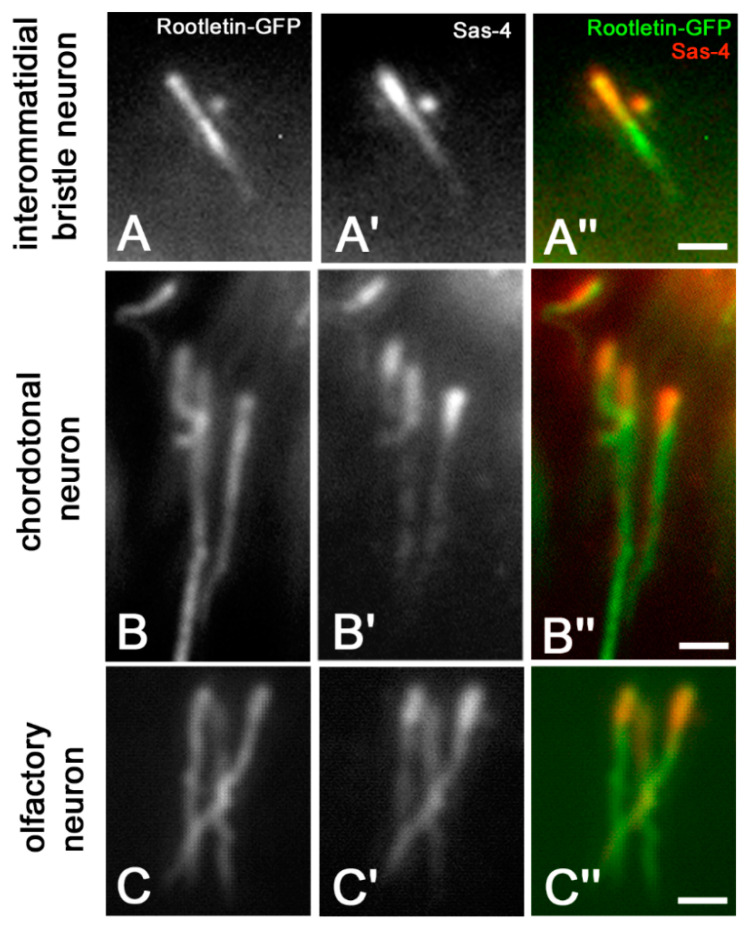
Colocalization of rootletin-GFP and Sas-4 in the interommatidial bristle (**A**–**A″**), chordotonal (**B**–**B″**) and olfactory (**C**–**C″**) neurons. Scale bars: 5 µm.

Since the bright Sas-4 filaments remember the shape and the position of the ciliary rootlets, we used a *Drosophila* strain that expressed rootletin, the main rootlet component, conjugated with GFP [7] to verify if there was a colocalization of these proteins. We find, indeed, that the Sas-4 staining colocalized with rootletin of the interommatidial bristles (Figure 6A–A″), and of chordotonal (Figure 6B–B″) and olfactory (Figure 6C–C″) neurons.

**Figure 7:**
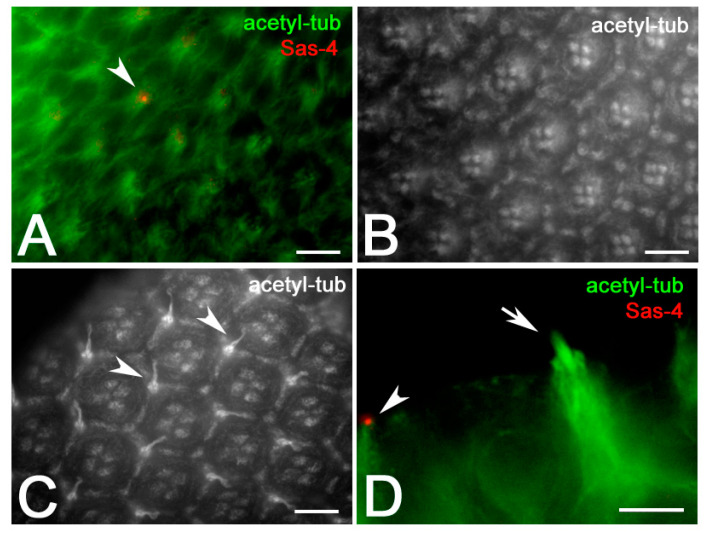
Organization of the pupal retina in *Sas-4* mutants. (**A**) Only one centriole (arrowhead) is seen within the ommatidial cells of this young pupal imaginal disc. Surface views of the pupal retina at 25 h (**B**) and 45 h (**C**) APF: the ommatidia have an overall wild-type organization and distinct mechanosensory bristles are visible at the apex of each ommatidial unit (arrowheads). (**D**) Lateral view of a mechanosensory bristle (arrow): the only centriole observed within this field (arrowhead) is far from the bristle. Scale bars: 10 µm (**A**–**C**), 2.5 µm (**D**).

It has been shown that the Sas-4 gene product is dispensable for the proper assembly of the *Drosophila* eye [28]. Centrioles were, indeed, rarely found in the eye imaginal discs of *Sas-4* mutants (Figure 7A). However, the overall organization of the developing ommatidia (Figure 7B) is like that observed in wild-type imaginal discs (Figure 1A). Moreover, the eye imaginal discs of *Sas-4* mutant pupae at 45 h AFP displayed distinct interommatidial bristles (Figure 7C) filled by longitudinal bundles of microtubules but the centrioles were lacking (Figure 7D).

## 4. Discussion

Our observations of the interommatidial mechanosensory bristles showed that Sas-4 is localized, as expected, on distal and proximal centrioles. However, we also find an unexpected filamentous localization that extends from the centrioles towards the basal region of the cell. A such ectopic localization of Sas-4 was also found in association with the tandemly aligned centrioles associated with the ciliary projections of chordotonal and olfactory neurons in the second and third antennal segments. Since we used a monoclonal antibody, we cannot exclude the possibility that the ectopic localization of Sas-4 could be attributed to a cross-reactivity of the antibody. However, we achieved the same results using a fly strain expressing a Sas-4-GFP. Therefore, the labeling we observed cannot be due to cross-reactivity of our antibody but rather identifies a new localization of Sas-4 that is not restricted to the centriole wall. 

To our knowledge, only two reports deal with a cytosolic distribution of Sas-4 independently by the centrioles. It has been observed that several proteins that organize the pericentriolar material (PCM) are recruited and translocated to the mother centrioles in the early *Drosophila* embryos as part of cytoplasmic complexes that contain Sas-4 [30]. Moreover, Sas-4 was also found in association with thin microtubule-based structures transiently present in the peripheral cytoplasm of early polar spermatocytes [38]. Remarkably, these transient structures emerge from only one of the two mother centrioles of the sister pair present in the germ cells, uncovering a new asymmetry between the parent centriole of the same pair. 

By using a *Drosophila* strain expressing rootletin-GFP, we also showed a distinct colocalization of Sas-4 with the ciliary rootlets of the interommatial mechanosensory cells and of the chordotonal and olfactory neurons. This observation raises questions about the meaning and/or the functional aspects, if any, of this ectopic Sas-4 localization. Different proteins are associated with the ciliary rootlets, in addition to rootletin, but the presence of Sas-4 was never reported. Since the formation of the ciliary rootlets depends on the presence of the centrioles [7] and the stability of the centriolar wall is linked to the action of Sas-4, we could attribute to Sas-4 a role in maintaining the stability of the rootlets and/or some functions in their assembly. 

Tandem aligned centrioles and distinct striated rootlets have been usually associated with the dendritic extension of the bipolar neurons in sensory organs. The finding of such structures in non-sensory cells represents, therefore, a remarkably exception. Interestingly, the distal centriole of the sheath cells is unable to nucleate a ciliary axoneme, suggesting that this property is exclusive of the sensory neurons and likely correlated with the presence of a dendritic extension. Moreover, the distal centriole of the sheath cells lacks Unc-GFP signal, a protein specifically expressed by *Drosophila* centrioles that nucleate a ciliary axoneme, such as spermatocyte and spermatid centrioles and the distal centrioles of the sensory organs [26]. The only exception to this specific localization is represented by evenly spaced Unc–GFP spots present in the eye imaginal discs of third instar larvae. These spots are restricted to the R8 photoreceptor cell of each ommatidium in association with mother centrioles that do not nucleate ciliary axonemes [5].

Since the centriole pairs and their associated rootlets do not seem to play functional roles in the absence of a dendritic process, we can speculate that their assembly is a default property of the secondary precursor cell that gives origin to sensory neurons and sheath cells. During the formation of the adult sensilla, a sensory organ precursor cell divides asymmetrically and generates two secondary precursors cells which in turn divide asymmetrically to give rise to two distinct pairs of differentiated cells: the neuron and the associated sheath cell and the trichogen and tormogen cells [39,40]. The divergent fate of the sister cells within each pair is essentially related to the different expression of Notch that also promotes neuron differentiation and supporting cell formation. Reduced Notch activity affects, indeed, the proper formation of the mechanosensory bristles [41]. Tandemly aligned centrioles have been also observed in the cytoplasm of the sheath cells in orthopteran sensilla [2] suggesting that this condition could represent a conserved trait of the insect sensory organs. However, such aligned centrioles have never been observed in the sheath cells of chordotonal and olfactory *Drosophila*. sensory neurons. 

## References

[1] Jana S.C., Mendonça S., Machado P., Werner S., Rocha J., Pereira A., Maiato H., Bettencourt-Dias M. (2018). Differential regulation of transition zone and centriole proteins contributes to ciliary base diversity. Nat. Cell Biol..

[2] Keil T.A. (2012). Sensory cilia in arthropods. Arthropod. Struct. Dev..

[3] Riparbelli M.G., Callaini G., Megraw T.L. (2012). Assembly and persistence of primary cilia in dividing *Drosophila* spermatocytes. Dev. Cell.

[4] Januschke J., Llamazares S., Reina J., Gonzalez C. (2011). *Drosophila* neuroblasts retain the daughter centrosome. Nat. Commun..

[5] Gottardo M., Callaini G., Riparbelli M.G. (2016). Does Unc–GFP uncover ciliary structures in the rhabdomeric eye of *Drosophila*?. J. Cell Sci..

[6] Gottardo M., Pollarolo G., Llamazares S., Reina J., Riparbelli M.G., Callaini G., Gonzalez C. (2015). Loss of Centrobin enables daughter centrioles to form sensory cilia in *Drosophila*. Curr. Biol..

[7] Chen J.V., Kao L.R., Jana S.C., Sivan-Loukianova E., Mendonça S., Cabrera O.A., Singh P., Cabernard C., Eberl D.F., Bettencourt-Dias M. (2015). Rootletin organizes the ciliary rootlet to achieve neuron sensory function in Drosophila. J. Cell Biol..

[8] Yang J., Liu X., Yue G., Adamian M., Bulgakov O., Li T. (2002). Rootletin, a novel coiled-coil protein, is a structural component of the ciliary rootlet. J. Cell Biol..

[9] Mohan S., Timbers T.A., Kennedy J., Blacque O.E., Leroux M.R. (2013). Striated rootlet and nonfilamentous forms of rootletin maintain ciliary function. Curr. Biol..

[10] Joukov V., De Nicolo A. (2019). The Centrosome and the Primary Cilium: The Yin and Yang of a Hybrid Organelle. Cells.

[11] Yang J., Gao J., Adamian M., Wen X.H., Pawlyk B., Zhang L., Sanderson M.J., Zuo J., Makino C.L., Li T. (2005). The ciliary rootlet maintains long-term stability of sensory cilia. Mol. Cell Biol..

[12] Styczynska-Soczka K., Jarman A.P. (2015). The *Drosophila* homologue of Rootletin is required for mechanosensory function and ciliary rootlet formation in chordotonal sensory neurons. Cilia.

[13] Ko D., Kim J., Rhee K., Choi H.J. (2020). Identification of a Structurally Dynamic Domain for Oligomer Formation in Rootletin. J. Mol. Biol..

[14] Vlijm R., Li X., Panic M., Rüthnick D., Hata S., Herrmannsdörfer F., Kuner T., Heilemann M., Engelhardt J., Hell S.W. (2018). STED nanoscopy of the centrosome linker reveals a CEP68-organized, periodic rootletin network anchored to a C-Nap1 ring at centrioles. Proc. Natl. Acad. Sci. USA.

[15] Cagan R.L., Ready D.F. (1989). The emergence of order in the Drosophila pupal retina. Dev. Biol..

[16] Lai E.C., Orgogozo V. (2004). A hidden program in Drosophila peripheral neurogenesis revealed: Fundamental principles underlying sensory organ diversity. Dev. Biol..

[17] Fu J., Glover D. (2016). How the newborn centriole becomes a mother. Cell Cycle.

[18] Zheng X., Gooi L.M., Wason A., Gabriel E., Mehrjardi N.Z., Yang Q., Zhang X., Debec A., Basiri M.L., Avidor-Reiss T. (2014). Conserved TCP domain of Sas-4/CPAP is essential for pericentriolar material tethering during centrosome biogenesis. Proc. Natl. Acad. Sci. USA.

[19] Ramani A., Mariappan A., Gottardo M., Mandad S., Urlaub H., Avidor-Reiss T., Riparbelli M., Callaini G., Debec A., Feederle R. (2018). Plk1/Polo Phosphorylates Sas-4 at the Onset of Mitosis for an Efficient Recruitment of Pericentriolar Material to Centrosomes. Cell Rep..

[20] Gabriel E., Wason A., Ramani A., Gooi L.M., Keller P., Pozniakovsky A., Poser I., Noack F., Telugu N.S., Calegari F. (2016). CPAP promotes timely cilium disassembly to maintain neural progenitor pool. EMBO J..

[21] Sharma A., Aher A., Dynes N.J., Frey D., Katrukha E.A., Jaussi R., Grigoriev I., Croisier M., Kammerer R.A., Akhmanova A. (2016). Centriolar CPAP/SAS-4 Imparts Slow Processive Microtubule Growth. Dev. Cell..

[22] Avidor-Reiss T., Gopalakrishnan J. (2013). Building a centriole. Curr. Opin. Cell Biol..

[23] Hatzopoulos G.N., Erat M.C., Cutts E., Rogala K.B., Slater L.M., Stansfeld P.J., Vakonakis I. (2013). Structural analysis of the G-box domain of the microcephaly protein CPAP suggests a role in centriole architecture. Structure.

[24] Gartenmann L., Wainman A., Qurashi M., Kaufmann R., Schubert S., Raff J.W., Dobbie I.M. (2017). A combined 3D-SIM/SMLM approach allows centriole proteins to be localized with a precision of ~4–5 nm. Curr. Biol..

[25] Varadarajan R., Rusan N.M. (2018). Bridging centrioles and PCM in proper space and time. Essays Biochem..

[26] Baker J.D., Adhikarakunnathu S., Kernan M.J. (2004). Mechanosensory-defective, male-sterile *unc* mutants identify a novel basal body protein required for ciliogenesis in *Drosophila*. Development.

[27] Zheng X., Ramani A., Soni K., Gottardo M., Zheng S., Ming Gooi L., Li W., Feng S., Mariappan A., Wason A. (2016). Molecular basis for CPAP-tubulin interaction in controlling centriolar and ciliary length. Nat. Commun..

[28] Basto R., Lau J., Vinogradova T., Gardiol A., Woods C.G., Khodjakov A., Raff J.W. (2006). Flies without centrioles. Cell.

[29] Lewis E. (1960). A new standard food medium. Dros. Inf. Service.

[30] Gopalakrishnan J., Mennella V., Blachon S., Zhai B., Smith A.H., Megraw T.L., Nicastro D., Gygi S.P., Agard D.A., Avidor-Reiss T. (2011). Sas-4 provides a scaffold for cytoplasmic complexes and tethers them in a centrosome. Nat. Commun..

[31] Reina J., Gottardo M., Riparbelli M.G., Llamazares S., Callaini G., Gonzalez C. (2018). Centrobin is essential for C-tubule assembly and flagellum development in *Drosophila* melanogaster spermatogenesis. J. Cell Biol..

[32] Rodrigues-Martins A., Bettencourt-Dias M., Riparbelli M., Ferreira C., Ferreira I., Callaini G., Glover D.M. (2007). DSAS-6 organizes a tube-like centriole precursor, and its absence suggests modularity in centriole assembly. Curr. Biol..

[33] Carthew R.W. (2007). Pattern formation in the *Drosophila* eye. Curr. Opin. Genet. Dev..

[34] Cagan R.L. (2009). Principles of *Drosophila* eye differentiation. Curr. Top. Dev. Biol..

[35] Perry M.M. (1968). Further studies on the development of the of Drosophila melanogaster. II. The interommatidial bristles. J. Morphol..

[36] Hsu W.B., Hung L.Y., Tang C.J., Su C.L., Chang Y., Tang T.K. (2008). Functional characterization of the microtubule-binding and -destabilizing domains of CPAP and d-SAS-4. Exp. Cell Res..

[37] Tang C.J., Fu R.H., Wu K.S., Hsu W.B., Tang T.K. (2009). CPAP is a cell-cycle regulated protein that controls centriole length. Nat. Cell Biol..

[38] Riparbelli M.G., Persico V., Callaini G. (2018). A transient microtubule-based structure uncovers a new intrinsic asymmetry between the mother centrioles in the early Drosophila spermatocytes. Cytoskeleton.

[39] Hartenstein V., Posakony J.W. (1989). Development of adult sensilla on the wing and notum of *Drosophila melanogaster*. Development.

[40] Schweisguth F. (2015). Asymmetric cell division in the *Drosophila* bristle lineage: From the polarization of sensory organ precursor cells to Notch-mediated binary fate decision. Wiley Interdiscip. Rev. Dev. Biol..

[41] Cagan R.L., Ready D.F. (1989). Notch is required for successive cell decisions in the developing *Drosophila* retina. Genes Dev..

